# Determinants of Urogenital Schistosomiasis Among Pregnant Women and its Association With Pregnancy Outcomes, Neonatal Deaths, and Child Growth

**DOI:** 10.1093/infdis/jiz664

**Published:** 2019-12-13

**Authors:** Wellington Murenjekwa, Rachel Makasi, Robert Ntozini, Bernard Chasekwa, Kuda Mutasa, Lawrence H Moulton, James M Tielsch, Jean H Humphrey, Laura E Smith, Andrew J Prendergast, Claire D Bourke

**Affiliations:** 1 Zvitambo Institute for Maternal and Child Health Research, Harare, Zimbabwe; 2 Department of International Health, Johns Hopkins Bloomberg School of Public Health, Baltimore, Maryland, USA; 3 Department of Global Health, Milken Institute School of Public Health, George Washington University, Washington, District of Columbia, USA; 4 Department of Epidemiology and Environmental Health, School of Public Health and Health Professions, University at Buffalo, Buffalo, New York, USA; 5 Blizard Institute, Queen Mary University of London, London, UK

**Keywords:** schistosomiasis, pregnancy, women, Zimbabwe, adverse birth outcomes, birthweight, stunting, *Schistosoma haematobium*, child health

## Abstract

**Background:**

*Schistosoma haematobium* is a parasitic helminth that causes urogenital pathology. The impact of urogenital schistosomiasis during pregnancy on birth outcomes and child growth is poorly understood.

**Methods:**

Risk factors for urogenital schistosomiasis were characterized among 4437 pregnant women enrolled in a cluster-randomized community-based trial in rural Zimbabwe. Infection was defined via urine microscopy (≥1 *S. haematobium* egg) and urinalysis (hematuria). Associations between infection and pregnancy outcomes were assessed in case-control analyses using conditional logistic regression. The association of maternal infection with birthweight and length-for-age Z scores (LAZ) at 1 and 18 months of age were assessed using generalized estimating equations.

**Results:**

Urogenital schistosomiasis (egg positive and/or hematuria positive) was detected in 26.8% of pregnant women. Risk factors significantly associated with infection were maternal age, education, marital status, and religion; household drinking water source and latrine; study region; and season. Urogenital schistosomiasis was not significantly associated with adverse pregnancy outcomes (miscarriage, stillbirth, preterm, and small-for-gestational age), birthweight, neonatal death, or LAZ.

**Conclusions:**

Including pregnant women in antihelminthic treatment programs would benefit a large number of women in rural Zimbabwe. However, clearance of the low-intensity infections that predominate in this context is unlikely to have additive benefits for pregnancy outcomes or child growth.

**Clinical Trials Registration:**

NCT01824940.

## Teaser

Urogenital schistosomiasis is common among pregnant women in rural Zimbabwe (n = 4437 in this study), but infection was not associated with adverse pregnancy outcomes, neonatal deaths, birthweight, or child length-for-age Z scores at 1 or 18 months of age.

Urogenital schistosomiasis is a highly prevalent disease in sub-Saharan Africa caused by *Schistosoma haematobium* parasites. Infection is transmitted by freshwater-dwelling larval schistosomes, which penetrate the skin, migrate, and mature into long-lived adult worms residing in urogenital blood vessels. Adult worm pairs continuously produce eggs, which pass from blood to urine for excretion. Ongoing egg deposition drives chronic tissue damage, which can progress to renal and urogenital dysfunction if untreated [1]. Microscopic detection of *S. haematobium* eggs in urine is diagnostic of urogenital schistosomiasis, which can also be identified indirectly via hematuria [2–5]. Adult worms can be cleared by the antihelminthic drug praziquantel; however, treatment does not clear immature parasites nor prevent reinfection [1]. Repeated exposure to new infections, which is common in endemic communities reliant on unprotected water sources, can drive high-intensity infections associated with more severe pathology [1]. International guidelines advocate for improved water, sanitation, and hygiene (WASH) provision and practice uptake as a means of controlling schistosomiasis [6].

Schistosome prevalence and infection intensity peak in school-age children, the predominant target of epidemiological evaluation and mass drug administration programs (MDA) [7, 8]. Much less is known about the risk factors for urogenital schistosomiasis among pregnant women. An estimated 40 million reproductive-age women are currently infected and 10 million African women per year have schistosomiasis during pregnancy [9]. Female genital schistosomiasis, which affects approximately 16 million women [10], includes active infections and genital pathology that persists posttreatment [11]. The World Health Organization (WHO) recommends inclusion of pregnant women in MDA [12]. Despite evidence from retrospective studies and randomized controlled trials that praziquantel treatment during pregnancy safely and effectively improves maternal health without adverse effects on the fetus [13–15], pregnant women continue to be excluded from schistosomiasis control programs in several countries [16], including Zimbabwe. It is hypothesized that schistosomiasis during pregnancy could lead to adverse birth outcomes and negatively impact child growth in early life [7, 9], which are associated with life-long health deficits [7, 17]. The prevalence of preterm birth in Zimbabwe is among the highest in the world [18] and 35% of children in rural Zimbabwe are stunted by 18 months of age [19]; it is unclear whether in utero exposure to urogenital schistosomiasis contributes to these conditions.

We surveyed urogenital schistosomiasis among pregnant women in rural Zimbabwe, assessed risk factors for infection, and monitored pregnancy outcomes, neonatal deaths, birthweight, and child linear growth. We hypothesized that maternal schistosomiasis would be associated with adverse pregnancy outcomes, low birthweight, and child stunting.

## METHODS

### Ethics

The Sanitation Hygiene Infant Nutrition Efficacy (SHINE) trial was approved by the Medical Research Council of Zimbabwe and the Institutional Review Board of the Johns Hopkins Bloomberg School of Public Health (Clinical Trials Registration: NCT01824940; full protocol: https://osf.io/w93hy). All women provided written informed consent to participate.

### Study Design and Setting

SHINE was a cluster-randomized community-based 2 × 2 factorial trial testing the independent and combined effects of a household WASH intervention and an infant and young child feeding (IYCF) intervention on child linear growth and hemoglobin at 18 months of age [20]; primary trial results are reported elsewhere [19, 21]. The study area comprised 2 rural districts of Midlands province, Zimbabwe (Shurugwi and Chirumanzu), which had high *S. haematobium* prevalence but low *S. mansoni* (intestinal schistosomiasis) and soil-transmitted helminth prevalence among school-age children [8]. Districts were divided into 212 clusters, defined as the catchment area of 1–4 village health workers employed by the Zimbabwean Ministry of Health and Child Care, which were randomized into 4 study arms (standard of care [SOC], IYCF, WASH, and IYCF + WASH). SHINE created 4 study offices (hubs): Mvuma and St Theresa in Chirumanzu district and Shurugwi town and Tongogara in Shurugwi district. National MDA provided praziquantel and albendazole to 5–15 year olds annually, in accordance with national guidelines.

Rainfall and temperature for each cluster were extracted from raster maps (October–March for rainfall and January–December for temperature; 1983–2015) obtained after kriging interpolation using 47 Zimbabwean weather stations.

### Study Population

Between 22 November 2012 and 27 March 2015, village health workers identified pregnancies through prospective surveillance; eligible women were permanent residents in the districts, confirmed pregnant, and provided written informed consent.

**Figure 1.**
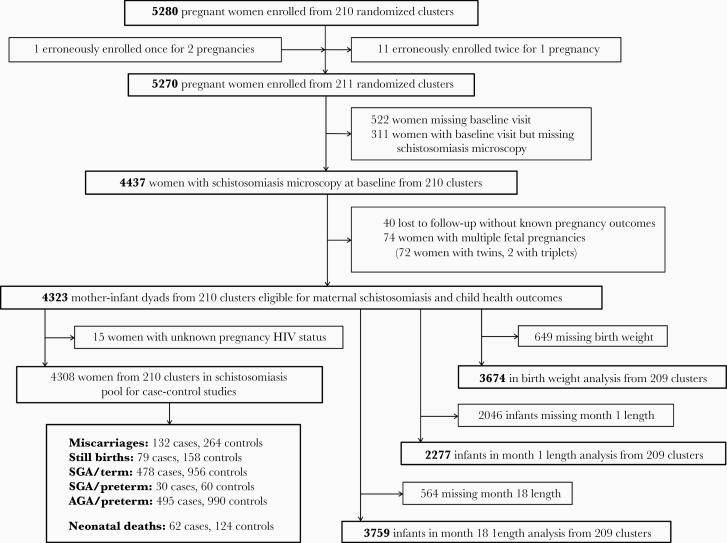
Selection of women for inclusion in assessment of risk factors for urogenital schistosomiasis during pregnancy and its impact on birth outcomes and child growth. In total, 212 clusters were randomized, 53 in each of the 4 trial arms (SOC, IYCF, WASH, and IYCF + WASH). After randomisation, 1 cluster was excluded because it was in an urban area, 1 was excluded because the village health workers covering it mainly had clients outside the study area, and 2 more were merged on the basis of subsequent data for village health worker coverage, leaving 210 clusters from the original randomisation. Three new cluster designations were created because of anomalies in the original mapping. For 2 of these clusters, the trial group was clear; the third contained areas that were in 2 trial groups, and was assigned to the under-represented group, resulting in 53 clusters in each group. All these changes occurred before enrolment began. When enrolment was completed, there was 1 cluster (SOC) in which no women were enrolled, leaving a total of 211 clusters available for analysis. Infant follow-up was lower at the 1-month than the 18-month visit due to cultural practice for women (especially primiparous) to return to their parental home during the perinatal period. Mothers of infants with 1-month LAZ were, on average, 1.5 years older and had higher parity than mothers of infants with missing data; no other meaningful differences were observed [22]. Of the 564 mother-infant dyads missing 18-month infant length, 146 were miscarriages, 86 stillbirths, 116 neonatal deaths, and 100 postnatal deaths (78 infant deaths, 11 child deaths, 7 deaths with unknown date of death, 2 deaths with unknown date of birth, and 2 deaths after 18 months). Abbreviations: AGA, appropriate for gestational age; IYCF, infant and young child feeding; SGA, small for gestational age; SOC, standard of care; WASH, water, sanitation, and hygiene.

Research nurses made home visits at baseline (approximately 2 weeks after mothers provided consent), 32 weeks’ gestation, and 1, 3, 6, 12, and 18 months postpartum to assess maternal and household characteristics and trial outcomes. Date of last menstrual period, height, weight, midupper arm circumference, hemoglobin concentrations (Hemocue), human immunodeficiency virus (HIV) status (rapid test algorithm Determine HIV-1/2 [Alere International Ltd] followed by INSTI HIV-1/2 [bioLytical Laboratories Inc.] if positive) and urogenital symptoms (questionnaire administered by research nurses) were determined at baseline. HIV-positive women were urged to seek immediate antenatal care for prevention of mother-to-child transmission. All participants with baseline parasitology data were included in urogenital schistosomiasis risk factor analyses (Figure 1).

### Urine Collection and Analysis

Participants provided a baseline urine sample; urinalysis was conducted immediately by a research nurse using Bayer Multistix 10 SG Reagent Strips. Women with abnormal results were referred to their local clinic. Women with any indication of hematuria on urinalysis (trace, small/+, moderate/++, or large/+++) were classified as hematuria positive. Urine aliquots (90mL) were transported to the nearest study hub for parasitology.

### Parasitology

*S. haematobium* eggs were quantified in a single urine sample by filtration and microscopy [23]. Urine (10 mL) was centrifuged (3000 rpm, 5 min) and the sediment incubated with 1 drop Lugol’s Iodine (15 s, ambient temperature). One drop of stained urine sediment was immediately microscopically examined (×40). Participants with ≥1 *S. haematobium* egg were classified egg positive and advised to seek treatment postdelivery. Infection intensity was reported as egg count/10mL urine and categorized as low (0–50 eggs/mL) or high (>50 eggs/mL).

### Monitoring Pregnancy Outcomes, Neonatal Deaths, and Child Growth

Fetal losses were reported to the study team. A narrative of the event was obtained by a research nurse and reviewed by a senior research nurse and the study physician. Based on the date of last menstrual period, fetal loss <28 gestational weeks was classified as miscarriage and fetal loss >28 gestational weeks as stillbirth.

For liveborn infants, delivery details and birthweight were transcribed from health facility records. SHINE provided Tanita BD-590 infant scales (Arlington Heights) to all 43 health institutions in the study area and trained staff in their use. Infants <2500 g were classified as low birthweight according to international standards [24]. Gestational age (GA) at delivery was calculated from the date of the mother’s last menstrual period. Preterm was defined as a liveborn infant with GA <37 completed weeks. Small for gestational age (SGA) was defined as birthweight <10th centile and appropriate for gestational age (AGA) as birthweight *>*10th centile [24]. Infants were categorized as SGA/term, SGA/preterm, AGA/term, and AGA/preterm. Neonatal deaths, defined as liveborn infants who died within 28 days of birth, were identified by village health workers/research nurses and reported to the study physician. Child length was compared to WHO growth standards to calculate length-for-age Z score (LAZ) and identify stunting (LAZ ≤ −2) [25]. Infants born to mothers with singleton pregnancies with known outcome and baseline parasitology were included in anthropometry analyses (Figure 1). Miscarriages, stillbirths, and neonatal deaths were reported to ethical review committees as serious adverse events.

### Case-Control Studies of Adverse Pregnancy Outcomes and Neonatal Deaths

Case-control analyses were conducted to evaluate the association between schistosomiasis during pregnancy and each adverse pregnancy outcome and neonatal death. Women who had singleton pregnancies with known outcome, known HIV status, known GA, and available baseline parasitology were eligible (Figure 1). For miscarriage, we used 1:2 incidence density sampling to select 1 woman who miscarried (case) and 2 women with the same HIV status, SHINE arm, and GA at baseline (±2 weeks) who did not miscarry by the gestational week of the case event (controls). We used a similar sampling approach for stillbirth, matching 1 woman who experienced fetal loss (case) to 2 women with the same HIV status, SHINE arm, GA at baseline (±2 weeks), and infant sex who did not have a stillbirth by the gestational week of the case event (controls). Controls were selected with replacement so that a single control could be matched to more than 1 case and could also become a case later. For neonatal deaths, we selected 1 woman whose liveborn infant died <28 days postpartum (case) and 2 women whose liveborn infant had not died by the time of the case event (controls), matched on maternal HIV status, SHINE arm, infant sex, and GA at baseline (±2 weeks). For SGA and preterm outcomes, we pooled the 3 case groups (SGA/term, AGA/preterm, and SGA/preterm), and matched AGA/term controls based on maternal HIV status, SHINE arm, infant sex, and GA at baseline (±2 weeks) until we had ≥1.73 times the number of controls in the largest case group. This was based on a group matching approach, ensuring that the minimum number of unique controls exceeded the largest case group by √k (k = number of case groups, 3).

### Statistical Analysis

Urogenital schistosomiasis was categorized into 3 diagnostic definitions: (1) *S. haematobium* egg-positive; (2) hematuria-positive; and (3) egg-positive and/or hematuria-positive. Cluster-adjusted *Χ*^2^ tests and 2-sided *t* tests were used to assess the relationship between schistosomiasis and categorical and continuous variables, respectively.

Generalized estimating equation (GEE) logistic regression with a logit link to estimate odds ratios and exchangeable correlation structure was used to model the relationship between egg or hematuria status and possible risk factors for infection identified from the literature. Because risk factors and infection were assessed at baseline, prior to SHINE interventions, analyses were not adjusted for SHINE arm. To accommodate the high frequency of egg-negative participants (89.4%), zero-inflated negative binomial regression (ZINB) was used to model the association between possible risk factors for infection and infection intensity. Variables significantly associated with egg and/or hematuria status at *P* < .25 were entered into multivariable GEE. Variables were entered into multivariable ZINB using the same criteria.

For case-control studies, separate conditional logistic regression models were used to model the association between schistosomiasis status and each adverse outcome. Models were adjusted for variables associated with both schistosomiasis and the outcome at *P* < .1; models for miscarriage, SGA/term, and SGA/preterm were adjusted for maternal age; models for stillbirth were adjusted for SHINE hub; and models for neonatal death and AGA/preterm were unadjusted. Separate unadjusted models were run for schistosome infection intensity and hematuria severity.

GEE population-averaged models accounting for clustering were used to model relationships between schistosomiasis and birthweight and 1-month and 18-month LAZ, and separately with the odds of low birthweight and 1-month and 18-month stunting. Models were adjusted for maternal age, maternal HIV status, and SHINE arm.

All statistical analyses were performed using STATA version 14.

## RESULTS

### Prevalence of Urogenital Schistosomiasis Among Pregnant Women

**Figure 2.**
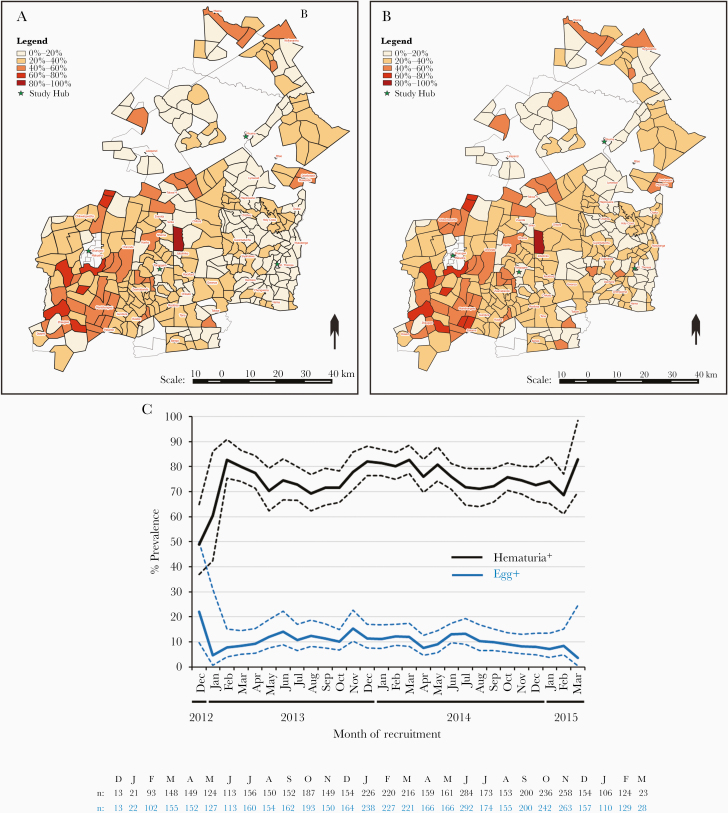
Prevalence of urogenital schistosomiasis among pregnant women in rural Zimbabwe by cluster and month of recruitment. The prevalence of (*A*) egg-positive women (n = 4437) and (*B*) hematuria-positive women (n = 4298) within each cluster (the catchment area of 1–4 village health workers). The 4 Sanitation Hygiene Infant Nutrition Efficacy (SHINE) study hubs (Mvuma, St. Theresa, Shurugwi, and Tongogara) are indicated by green stars; samples from each cluster were processed at the nearest hub. *C*, The percentage prevalence of egg-positive and hematuria-positive urine samples among pregnant women by month of recruitment to the SHINE study; frequency of participants with available parasitology and urinalysis data is indicated for each month under the graph. SHINE cluster-adjusted upper and lower 95% confidence intervals for prevalence estimates are indicated by dashed lines.

Of 5280 pregnant women enrolled in SHINE, 4437 with a median of 12.14 weeks’ gestation (interquartile range, 9.43–16.29) at baseline were included in this study (Table 1). Of these, 471 (10.6%) were egg-positive. Of the egg-positive women, 426 (90.4%) had low-intensity, 38 (8.1%) had high-intensity, and 7 (1.5%) had unknown-intensity infections. Of the 4298 women who also had urinalysis data, 1048 (24.4%) were hematuria-positive. Of the hematuria-positive women, 394 had trace (37.6%), 101 had small/+ (9.6%), 279 had moderate/++ (26.6%), and 274 had large/+++ hematuria (26.1%). Of the egg-positive women with urinalysis data, 79.0% were also hematuria-positive; women with more severe hematuria had higher infection intensity (Supplementary Figure 1). The distribution of egg-positive and hematuria-positive women by study cluster and enrolment month is shown in Figure 2. Overall, 1155 out of 4308 women (26.8%) with urinalysis data and/or who were egg-positive (ie, could be classified as infected without urinalysis data) had 1 or both indicators of urogenital schistosomiasis. More egg-positive women (33.3% of egg-positive vs 18.7% of egg-negative; *Χ*^2^, 54.0; *P* < .001) and hematuria-positive women (33.5% of hematuria-positive vs 16.1% hematuria-negative; *Χ*^2^, 147.3; *P* < .001) had detectable leukocytes in their urine indicative of urogenital inflammation. Urogenital symptoms were infrequent; however, significantly higher proportions of egg-positive and/or hematuria-positive women reported lower abdominal pain and hemoglobin levels were significantly lower among hematuria-positive and egg-positive and/or hematuria-positive women (Supplementary Table 1). Proportions of HIV-positive women did not differ according to schistosome infection status (Supplementary Table 1).

**Table 1.** Characteristics of Women Enrolled in the Study of Risk Factors for Urogenital Schistosomiasis During Pregnancy and Effects on Birth and Child Health Outcomes

Characteristic (n = 4437^a^)	Value
Maternal factors	
Parity, median (IQR)	2 (1–3)
Years of education, median (IQR)	10 (9–11)
Age of mother, y, median (IQR)	25.5 (20.5–31.2)
Age category,^b^ n (%)	
Above 15 y	4165 (99.5)
15 y and younger	22 (0.5)
Employed, n (%)	
Yes	379 (8.7)
No	3973 (91.3)
Married, n (%)	
Yes	4008 (95.5)
No	188 (4.5)
Religion, n (%)	
Apostolic	1967 (46.6)
Other Christians^c^	1902 (45.0)
Other religions	355 (8.4)
HIV status, n (%)	
Positive	732 (16.5)
Negative	3687 (83.1)
Unknown	18 (0.4)
Household factors	
Household wealth, n (%)	
Lowest quintile	877 (20.1)
Second quintile	878 (20.1)
Middle quintile	875 (20.1)
Fourth quintile	866 (19.8)
Highest quintile	869 (19.9)
Household size, median (IQR)	5 (3–6)
WASH factors	
Median 1-way walk time to fetch water, min, median (IQR)	10 (5–20)
Drinking water, n (%)	
Improved	2703 (62.7)
Not improved	1605 (37.3)
Any latrine, n (%)	
Yes	1548 (36.0)
No	2755 (64.0)
Improved latrine, n (%)	
Yes	1355 (31.5)
No	2943 (68.5)
Handwashing station, n (%)	
Yes	376 (9.2)
No	3702 (90.8)
Water available at handwashing station, n (%)	
Yes	126 (3.1)
No	3942 (96.9)
Environmental factors	
Minimum temperature, °C, median (IQR)	11.7 (11.6–11.8)
Maximum temperature, °C, median (IQR)	25.8 (25.6–25.9)
Mean rainfall, mm, median (IQR)	595.9 (587.4–602.7)
Season at enrolment, n (%)	
Rainy	1581 (35.8)
Cold	1352 (30.6)
Hot and dry	1488 (33.7)
Field office/hub, n (%)	
Chirumanzu district	
Mvuma	983 (22.2)
St Theresa	1096 (24.7)
Shurugwi district	
Shurugwi	1416 (31.9)
Tongogara	942 (21.2)

Abbreviation: HIV, human immunodeficiency virus; IQR, interquartile range; SHINE, Sanitation Hygiene Infant Nutrition Efficacy Study; WASH, water, sanitation, and hygiene.

^a^All women enrolled in SHINE with baseline urine microscopy to detect *Schistosoma haematobium* eggs, known HIV status, and known pregnancy outcome.

^b^Children aged 5–15 years are eligible for inclusion in Zimbabwean national mass antihelminthic drug administration programs, which were conducted annually in SHINE study districts. Pregnant girls are excluded from the mass drug administration programs.

^c^Other Christians include: Protestants, Pentecostals, Catholics, and other Christian groups.

### Risk Factors for Urogenital Schistosomiasis Among Pregnant Women

Odds of being egg-positive and hematuria-positive were significantly lower in older women and among those with more years of education (Table 2 and univariable analysis in Supplementary Table 2). Limited access to household WASH at baseline was associated with greater odds of being egg-positive (no improved drinking water) and hematuria-positive (no improved latrine and no improved drinking water; Table 2). Women recruited from Shurugwi hub had significantly higher odds of being egg-positive and hematuria-positive than women from Mvuma (Table 2). Women enrolled and tested for schistosomiasis during the hot and dry season had significantly higher odds of being hematuria-positive than those recruited during the rainy season (Table 2).

**Table 2.** Multivariable Generalized Estimating Equation Logistic Regression Analysis of Risk Factors for Being *Schistosoma haematobium* Egg-Positive or Hematuria-Positive Among Pregnant Women

Factor	Multivariable GEE Logistic Regression Model			
	*S. haematobium* Egg-Positive (n = 4437)		Hematuria-Positive (n = 4298)	
	Adjusted OR^a,b^ (95% CI)	*P*	Adjusted OR^a,c^ (95% CI)	*P*
Maternal factors				
Education	0.94 (.89–1.00)	.034	0.94 (.90–.98)	.003
Age of mother	0.94 (.92– .96)	<.001	0.97 (.95–.98)	<.001
Religion				
Apostolic	1.00			
** ** Other Christians^d^	0.82 (.64– 1.05)	.115		
** ** Other religions	1.03 (.72– 1.46)	.886		
WASH factors				
Improved latrine				
** ** Yes			1.00	
** ** No			1.30 (1.10–1.54)	.002
Improved drinking water				
** ** Yes	1.00		1.00	
** ** No	1.27 (1.03– 1.57)	.023	1.21 (1.02–1.44)	.029
Environmental factors				
Study hub				
Chirumanzu district				
** ** Mvuma	1.00		1.00	
** ** St Theresa	1.23 (.84–1.78)	.284	0.80 (.62–1.04)	.098
Shurugwi district				
** ** Shurugwi	1.68 (1.22–2.30)	.001	1.99 (1.53–2.57)	<.001
** ** Tongogara	1.24 (.87–1.76)	.234	1.20 (.92–1.57)	.169
Season at enrolment				
** ** Rainy			1.00	
** ** Cold			1.19 (.99–1.44)	.067
** ** Hot and dry			1.38 (1.14–1.67)	.001

Abbreviations: CI, confidence interval; GEE, generalized estimating equation; OR, odds ratio; WASH, water, sanitation, and hygiene.

^a^Adjusted for: all other factors included in the multivariable model; variables associated with egg and/or hematuria status at *P* < .25 in univariable GEE (Supplementary Table 2) were entered into multivariable GEE.

^b^Odds of being positive for ≥1 *S. haematobium* egg per 10mL urine.

^c^Odds of being positive for hematuria.

^d^Other Christians include: Protestants, Pentecostals, Catholics, and other Christian groups.

Multivariable ZINB, which models egg count rather than status, also identified maternal age and study hub as significant risk factors for being egg positive (Table 3 and univariable analysis in Supplementary Table 3). Compared to the Apostolic faith group, women of other Christian faiths had greater odds of being egg negative (ie, lower odds of infection; Table 3). Of the egg-positive women, unmarried women had higher-intensity infections than married women (Table 3). Despite having lower odds of being egg-negative, egg-positive women from Shurugwi hub had lower-intensity infections than egg-positive women from Mvuma (Table 3).

**Table 3.** Multivariable Zero-Inflated β Regression Analysis of Risk Factors for High *Schistosoma haematobium* Infection Intensity Among Pregnant Women

Factor	Zero-Inflated Negative Binomial Model			
	Odds of Egg Negative (n = 4437)		Infection Intensity (n = 4430^a^)	
	Adjusted OR^b, c^ (95% CI)	*P*	IRR (95% CI)	*P*
Maternal factors				
Parity	0.99 (.85–1.15)	.888	0.95 (.82–1.10)	.483
Years of education	1.06 (.98–1.14)	.166		
Age	1.07 (1.03–1.11)	.001		
Marital status				
Married	1.00		1.00	
Not married	1.52 (.55–4.22)	.420	2.91 (1.06–8.04)	.039
Religion				
Apostolic	1.00		1.00	
Other Christians^d^	1.45 (1.04–2.04)	.031	1.41 (.93–2.14)	.104
Other religions	0.97 (.56–1.69)	.914	1.14 (.68–1.91)	.614
Household factors				
Household wealth				
Lowest quintile	1.00			
Second	0.93 (.61–1.41)	.720		
Middle	1.36 (.84–2.20)	.207		
Fourth	1.16 (.75–1.79)	.498		
Highest quintile	1.23 (.70–2.18)	.476		
WASH factors				
Any latrine				
Yes	1.00			
No	0.83 (.41–1.69)	.606		
Improved latrine				
Yes	1.00			
No	1.07 (.51–2.22)	.863		
Improved drinking water				
Yes	1.00			
No	0.81 (.61–1.07)	.143		
Environmental factors				
Minimum temperature			0.79 (.21–2.91)	.722
Study hub				
Chirumanzu district				
Mvuma	1.00			
St Theresa	0.77 (.47–1.25)	.285	0.75 (.39–1.44)	.384
Shurugwi district				
Shurugwi	0.48 (.30–.75)	.001	0.45 (.25–.80)	.007
Tongogara	0.73 (.45–1.20)	.217	1.04 (.50–2.21)	.891

Abbreviations: CI, confidence interval; IRR, incidence risk ratio; OR, odds ratio; WASH, water, sanitation, and hygiene.

^a^Seven participants had known schistosomiasis status but unknown infection intensity.

^b^Adjusted for all other factors included in the multivariable model; variables associated with infection at *P* < .25 in univariable GEE (Supplementary Table 3) were entered into multivariable models.

^c^Odds of being negative for *S. haematobium* eggs in urine.

^d^Other Christians include: Protestants, Pentecostals, Catholics, and other Christian groups.

### Urogenital Schistosomiasis, Pregnancy Outcomes, and Neonatal Death

Associations between urogenital schistosomiasis status, adverse pregnancy outcomes, and neonatal deaths are shown in Table 4. A higher percentage of SGA/preterm cases had egg-positive mothers than controls (egg positive 20.0% vs egg negative 5.0%; Supplementary Table 4; *P* < .05 in unadjusted GEE; Table 4). However, this was not significant in adjusted analysis and there was no significant association between schistosomiasis during pregnancy and any of the other adverse pregnancy outcomes or neonatal death (Table 4). There was also no significant association between infection intensity and any of the adverse birth outcomes (Supplementary Tables 5). There was some evidence for an association between AGA preterm and moderate/++ hematuria among the 15 cases in this category; however, large/+++ hematuria was not associated with AGA preterm (Supplementary Table 6).

**Table 4.** Effect of Maternal Urogenital Schistosomiasis During Pregnancy on Adverse Pregnancy Outcomes and Neonatal Deaths

Adverse outcome^a^	Egg-Positive				Hematuria-Positive				Egg and/or Hematuria-Positive			
	Unadjusted OR (95% CI)	*P*	Adjusted OR (95% CI)	*P*	Unadjusted OR (95% CI)	*P*	Adjusted OR (95% CI)	*P*	Unadjusted OR (95% CI)	*P*	Adjusted OR (95% CI)	*P*
Miscarriage^b^	0.75 (.39–1.45)	.395	0.80 (.40–1.57)	.513	0.73 (.45–1.18)	.195	0.76 (.46–1.26)	.291	0.75 (.47–1.20)	.234	0.79 (.49–1.29)	.348
Stillbirth^c^	0.71 (.22–2.32)	.572	0.67 (.20–2.23)	.519	1.06 (.55–2.01)	.869	1.11 (.57–2.16)	.757	1.09 (.59–2.01)	.792	1.12 (.60–2.10)	.713
SGA term^b^	1.30 (.92–1.84)	.144	1.28 (.88–1.85)	.195	1.20 (.94–1.54)	.153	1.12 (.86–1.45)	.402	1.13 (.89–1.44)	.320	1.07 (.83–1.38)	.599
SGA preterm^b^	5.31 (1.05–26.77)	.043	3.14 (.58–17.05)	.185	1.63 (.57–4.69)	.365	2.06 (.60–7.13)	.252	2.50 (.81–7.67)	.109	3.16 (.81–12.24)	.097
AGA preterm^d^	0.93 (.66–1.29)	.654			0.86 (.66–1.12)	.262			0.84 (.65–1.09)	.181		
Neonatal death^d^	0.77 (.28–2.13)	.618			0.72 (.32–1.60)	.413			0.84 (.39–1.77)	.639		

Abbreviations: AGA, appropriate for gestational age; CI, confidence interval; OR, odds ratio; SGA, small for gestational age.

^a^All cases and controls were matched on maternal HIV status, gestational age at the baseline visit (±2 weeks) and SHINE study arm; for stillbirth, neonatal death, SGA and preterm outcomes, cases and controls were also matched on infant sex.

^b^Adjusted for maternal age.

^c^Adjusted for field office/hub.

^d^Unadjusted.

### Urogenital Schistosomiasis and Birthweight

Of 3674 children with available data, 301 (8.2%) had low birthweight. There was no significant association between maternal schistosomiasis status, infection intensity, or hematuria severity and birthweight or the odds of low birthweight (Table 5 and Supplementary Tables 7 and 8).

**Table 5.** Effect of Urogenital Schistosomiasis During Pregnancy on Birthweight and Postnatal Child Linear Growth

Health Outcome	Egg-Positive				Hematuria-Positive				Egg and/or Hematuria-Positive			
	Unadjusted Coeff (95% CI)	*P*	Adjusted Coeff^a^ (95% CI)	*P*	Unadjusted Coeff (95% CI)	*P*	Adjusted Coeff^a^ (95% CI)	*P*	Unadjusted Coeff (95% CI)	*P*	Adjusted Coeff^a^ (95% CI)	*P*
Birthweight, g	−0.05 (−.01 to .02)	.060	−0.04 (−.09 to .01)	.090	−0.01 (−.05 to .02)	.452	0.00 (−.04 to .03)	.835	−0.02 (−.05 to .02)	.353	−0.01 (−.04 to .03)	.741
1-month LAZ	0.12 (−.08 to .32)	.254	0.12 (−.09 to .33)	.250	−0.06 (−.20 to .07)	.359	−0.06 (−.20 to .08)	.379	−0.03 (−.17 to .10)	.618	−0.03 (−.16 to .11)	.709
18-month LAZ	−0.07 (−.18 to .05)	.263	−0.04 (−.16 to .09)	.563	−0.05 (−.14 to .04)	.277	−0.01 (−.10 to .08)	.851	−0.05 (−.14 to .03)	.239	−0.02 (−.10 to .07)	.716
	Unadjusted OR (95% CI)	*P*	Adjusted OR^a^ (95% CI)	*P*	Unadjusted OR (95% CI)	*P*	Adjusted OR^a^ (95% CI)	*P*	Unadjusted OR (95% CI)	*P*	Adjusted OR^a^ (95% CI)	*P*
Low birthweight	1.32 (.91–1.92)	.145	1.26 (.85–1.86)	.258	1.08 (.79–1.48)	.613	1.00 (.72–1.38)	.978	1.13 (.84–1.51)	.433	1.04 (.77–1.42)	.781
1-month stunted	0.72 (.45–1.14)	.162	0.69 (.43–1.11)	.124	0.99 (.74–1.33)	.958	1.00 (.74–1.35)	.990	1.00 (.75–1.32)	.975	0.99 (.74–1.33)	.944
18-month stunted	1.03 (.81–1.31)	.794	0.97 (.75–1.25)	.799	1.08 (.90–1.30)	.422	1.00 (.83–1.19)	.930	1.09 (.92–1.30)	.303	1.02 (.86–1.21)	.844

Abbreviations: CI, confidence interval; Coeff, coefficient; IYCF, infant and young child feeding; LAZ, length-for-age Z scores; OR, odds ratio; WASH, water, sanitation, and hygiene.

^a^Adjusted for maternal age, maternal HIV status, and Sanitation Hygiene Infant Nutrition Efficacy (SHINE) study arm (standard of care, IYCF, WASH, and IYCF + WASH).

### Urogenital Schistosomiasis and Child Linear Growth

Anthropometry was available for 2277 children at 1 month and 3764 children at 18 months; 371 (16.3%) and 1212 (32.2%) children were stunted at these timepoints. Maternal schistosomiasis status, intensity, and hematuria severity were not significantly associated with LAZ or the odds of child stunting at 1 or 18 months (Table 5 and Supplementary Tables 7 and 8).

## DISCUSSION

We identified a high prevalence of urogenital schistosomiasis among pregnant women in rural Zimbabwe, via *S. haematobium* eggs in urine (10.6%), hematuria (24.4%), or both/either indicator (26.8%). Infections were predominantly low intensity, trace-to-moderate hematuria, and without urogenital symptoms. Infection was associated with younger age, fewer years of education, poor WASH access, religious practice, region, and season of recruitment, consistent with other demographic groups in Zimbabwe [8, 11, 26]. Despite the substantial morbidity associated with chronic schistosomiasis [1, 7, 9], infection status during pregnancy was not associated with adverse pregnancy outcomes, neonatal death, low birthweight, or child stunting.

Hematuria was included in our definition of urogenital schistosomiasis to reflect the single urine sample available per participant, the low sensitivity of urine microscopy for low-intensity infections, which were predominant in our cohort, and female genital schistosomiasis pathology due to encystment of eggs in tissues [11]. Both eggs and hematuria have established validity for detection of urogenital schistosomiasis in endemic areas [2, 3, 5], although hematuria can result from alternative causes [27, 28]. The association between hematuria and urogenital schistosomiasis is supported in our cohort by the geographical overlap between egg-positive and hematuria-positive prevalence, and higher infection intensities among women with more severe hematuria.

Existing data on *S. haematobium* epidemiology in Zimbabwe primarily come from school-age children [8]. We demonstrate that pregnant women share similar risk factors for schistosomiasis, despite the high percentage of Zimbabwean women with awareness of transmission and prevention methods [29]. Maternal age was significantly associated with schistosomiasis; younger women had greater odds of being egg positive or hematuria positive, consistent with previous studies [30–32]. A convex age-prevalence and age-infection intensity relationship is typical in schistosome-endemic communities, with infections accumulated in early life, peaking in childhood, and declining with age thereafter [33]. The age-related decline in infection could be driven by development of immune-mediated resistance [33, 34], increasing years of education [29], and/or changes in physiology [35, 36] and water contact behavior [2, 37–39]. For example, younger women tend to have more water contacts than older women [38] and a higher percentage of older women are aware of schistosomiasis [29]. Consistent with household WASH influencing transmission, odds of being infected were higher among women without an improved latrine or improved drinking water. Apostolic Christians, known for using unprotected water sources during religious practices [29], were more likely to be infected than other Christian groups. Women recruited in the dry season had greater odds of being hematuria positive than those recruited during the rainy season; however, because schistosomes are long lived, the season of initial infection could not be ascertained. Before MDA, Midlands province was in the highest prevalence and intensity categories for *S. haematobium* infection among school-age children in Zimbabwe [8]. We show that urogenital schistosomiasis prevalence is also high among pregnant women in Midlands province, and Shurugwi district in particular. Women from Shurugwi hub had the highest odds of infection; however, egg-positive women from Shurugwi hub had lower infection intensities than egg-positive women from Mvuma. Such regional differences may be explained by prioritization of Shurugwi for MDA among school children, as advocated by 2014 surveys [8], and/or development of immunity to new infections at a younger age in Shurugwi driven by higher local exposure rates [33, 34].

Urogenital inflammation due to schistosomiasis has been hypothesized to drive adverse birth outcomes [7, 9]. However, we did not find evidence that schistosomiasis status affected adverse pregnancy outcomes, neonatal death, birthweight, or LAZ. Consistent with our data, being *S. haematobium* egg positive was not associated with preterm among Gabonese women; in contrast, infection was significantly associated with low birthweight [32]. Infection prevalence was similar in the 2 cohorts (9% vs 10.6% egg positive); however, differences in the association between schistosomiasis and birthweight may reflect distinct *P. falciparum* malaria prevalences (a risk factor for low birthweight [32]), study designs, and diagnostic approaches. Prior studies of *S. haematobium* and *S. japonicum* identified negative associations between being egg positive during pregnancy and birthweight [40, 41]. However, the former study only recruited women with severe urogenital symptoms and the latter lacked adjustment for baseline case-control differences [9]. Our study builds on insights from existing studies with a considerably larger cohort of women and more comprehensive adjustment for confounders and is among the first to assess the relationship between hematuria and birth outcomes.

International guidelines recommend praziquantel treatment during pregnancy [12], which reduces schistosome-driven morbidity and mortality for women [13, 14]. Over a quarter of the pregnant women we tested had urogenital schistosomiasis, supporting their inclusion in national MDA. However, we did not identify an association between schistosomiasis and child health outcomes. Our data suggest that praziquantel treatment during pregnancy may not have additive benefits on pregnancy outcomes and child growth, at least in regions such as rural Zimbabwe where maternal infections are predominantly low intensity and associated with trace-to-moderate hematuria and few urogenital symptoms. This is supported by randomized controlled trials of praziquantel treatment among pregnant women for *S. mansoni* and *S. japonicum* after the first trimester [13, 14]; infection clearance was effective and safe for women without a negative impact on their infants, but did not improve birth outcomes or birthweight relative to placebo [13, 14]. Trials of praziquantel treatment earlier during pregnancy and in regions, such as Zimbabwe, predominantly affected by urogenital rather than intestinal schistosomiasis, could add to the existing body of evidence supporting treatment of schistosomiasis during pregnancy. Praziquantel treatment among cohorts of pregnant women with higher-intensity infections could plausibly have a greater impact on birth outcomes and child growth and/or alternative child health benefits to those that we assessed.

The strengths of our study were its large cohort size, comprehensive multivariable risk factor analysis, case-control matching for adverse birth outcomes with adjustment for confounders, child growth follow-up, and 3 diagnostic definitions for infection. Limitations included a single baseline urine sample during early pregnancy, which may have reduced diagnostic sensitivity and missed infections in later pregnancy [8]; potential interobserver microscopy differences between hubs; lack of maternal praziquantel treatment history and prepregnancy maternal weight, a predictor of adverse birth outcomes and stunting; no female genital schistosomiasis examination [10, 42]; and, although we present supplementary analyses of associations between infection intensity or hematuria severity, pregnancy outcomes and child growth using available data, these are underpowered.

Adverse birth outcomes and child growth deficits are very common in Zimbabwe [18, 19]. Our data show conclusively that, at least for women with low-intensity infections, urogenital schistosomiasis status during pregnancy is not associated with these outcomes. Although our data do not argue against the health benefits of praziquantel treatment for pregnant women, they suggest that treating low-intensity urogenital schistosomiasis is unlikely to avert adverse birth outcomes, neonatal death, low birthweight, or child stunting.

## Supplementary Data

Supplementary materials are available at *The Journal of Infectious Diseases* online. Consisting of data provided by the authors to benefit the reader, the posted materials are not copyedited and are the sole responsibility of the authors, so questions or comments should be addressed to the corresponding author.

jiz664_suppl_Supplementary_Material

jiz664_suppl_Supplementary_Figure_S1

## Notes

***Acknowledgments.*** We thank all the mothers, babies, and their families who participated in SHINE; the leadership and staff of the Ministry of Health and Child Care in Chirumanzu and Shurugwi districts and Midlands Province for operationalization of SHINE study procedures; the Ministry of Local Government officials for support and facilitation of SHINE field operations; Phillipa Rambanepasi and her team for financial management of SHINE; Virginia Sauramba for compliance management of SHINE; Dr Terence Mushore for extracting temperature and rainfall data; Dr Nicholas Midzi for useful discussions in advance of SHINE; and our program officers at the Bill and Melinda Gates Foundation and Department for International Development (UK Aid).

***Author contributions.*** W. M. led the statistical analysis of the urogenital schistosomiasis risk factor data from SHINE with assistance from R. M. B. C. conducted case-control analyses of adverse birth outcomes. R. N. developed and managed all information technology, data, and analysis for the SHINE trial. K. M. managed and implemented laboratory assays for the SHINE trial. L. H. M. was the SHINE senior statistician. J. M. T. contributed to design and interpretation of SHINE. J. H. H. was the principal investigator of SHINE. L. E. S. contributed to analysis and interpretation of SHINE. A. J. P. managed data collection and laboratory teams, and directed clinical and laboratory aspects of SHINE. C. D. B. managed the analysis, interpretation, and write-up of urogenital schistosomiasis data from SHINE. W. M. and C. D. B. prepared the manuscript. All authors reviewed and approved the manuscript for submission.

***Financial support.*** This work was supported by the Bill and Melinda Gates Foundation (grant numbers OPP1021542 to Johns Hopkins Bloomberg School of Public Health and OPP1143707 to Zvitambo Institute for Maternal and Child Health Research); the UK Department for International Development; the Wellcome Trust (grant numbers 108065/Z/15/Z to A. J. P. and 206225/Z/17/Z awarded together with the Royal Society to C. D. B.); the Swiss Agency for Development and Cooperation (grant number 8106727); UNICEF (grant number PCA-2017-0002); and the US National Institutes of Health (grant number R01 HD060338/HD/NICHD).

***Potential conflicts of interest.*** All authors: No reported conflicts of interest. All authors have submitted the ICMJE Form for Disclosure of Potential Conflicts of Interest. Conflicts that the editors consider relevant to the content of the manuscript have been disclosed.
