# Supplementary material for: Determinants of Urogenital Schistosomiasis Among Pregnant Women and its Association With Pregnancy Outcomes, Neonatal Deaths, and Child Growth
Source: J Infect Dis. 2019 Dec 13;223(8):1433–44. doi: 10.1093/infdis/jiz664 (PMC8064048; doi:10.1093/infdis/jiz664)
Supplement: jiz664_suppl_Supplementary_Material [file jiz664_suppl_supplementary_material.docx]

**SUPPLEMENTARY MATERIALS**

**Supplementary Figure 1:** *Schistosoma haematobium* infection intensity by hematuria category among pregnant women

**Supplementary Table 1:** Percentage of pregnant women with urogenital symptoms, anaemia and/or HIV

**Supplementary Table 2:** Univariable general estimating equation logistic regression analysis of risk

factors for being *S. haematobium* egg-positive or hematuria-positive among pregnant women

**Supplementary Table 3:** Univariable zero-inflated beta regression analysis of risk factors for high *S. haematobium* infection intensity among pregnant women

**Supplementary Table 4:** Prevalence of urogenital schistosomiasis in the case-control study of adverse birth outcomes among pregnant women

**Supplementary Tables 5**: Unadjusted association between maternal *S. haematobium* infection intensity during pregnancy and adverse birth outcomes

**Supplementary Tables 6:** Unadjusted association between the severity of maternal hematuria during pregnancy and adverse birth outcomes

**Supplementary Tables 7:** Unadjusted association between maternal *S. haematobium* infection intensity during pregnancy and child growth outcomes

**Supplementary Tables 8:** Unadjusted association between the severity of maternal hematuria during pregnancy and child growth outcomes

**Supplementary Figure 1: *Schistosoma haematobium* infection intensity by haematuria category among pregnant women.** Infection intensity (eggs/10mL) was compared across categories of hematuria severity determined by Multistix 10SG Urinalysis Test Strips using Kruskall Wallis test (test statistic:1237, p<0.001; n=4194). Infection intensity of women in each hematuria-positive category was then compared relative to hematuria-negative women by uncorrected Dunn’s test; ***p<0.001.


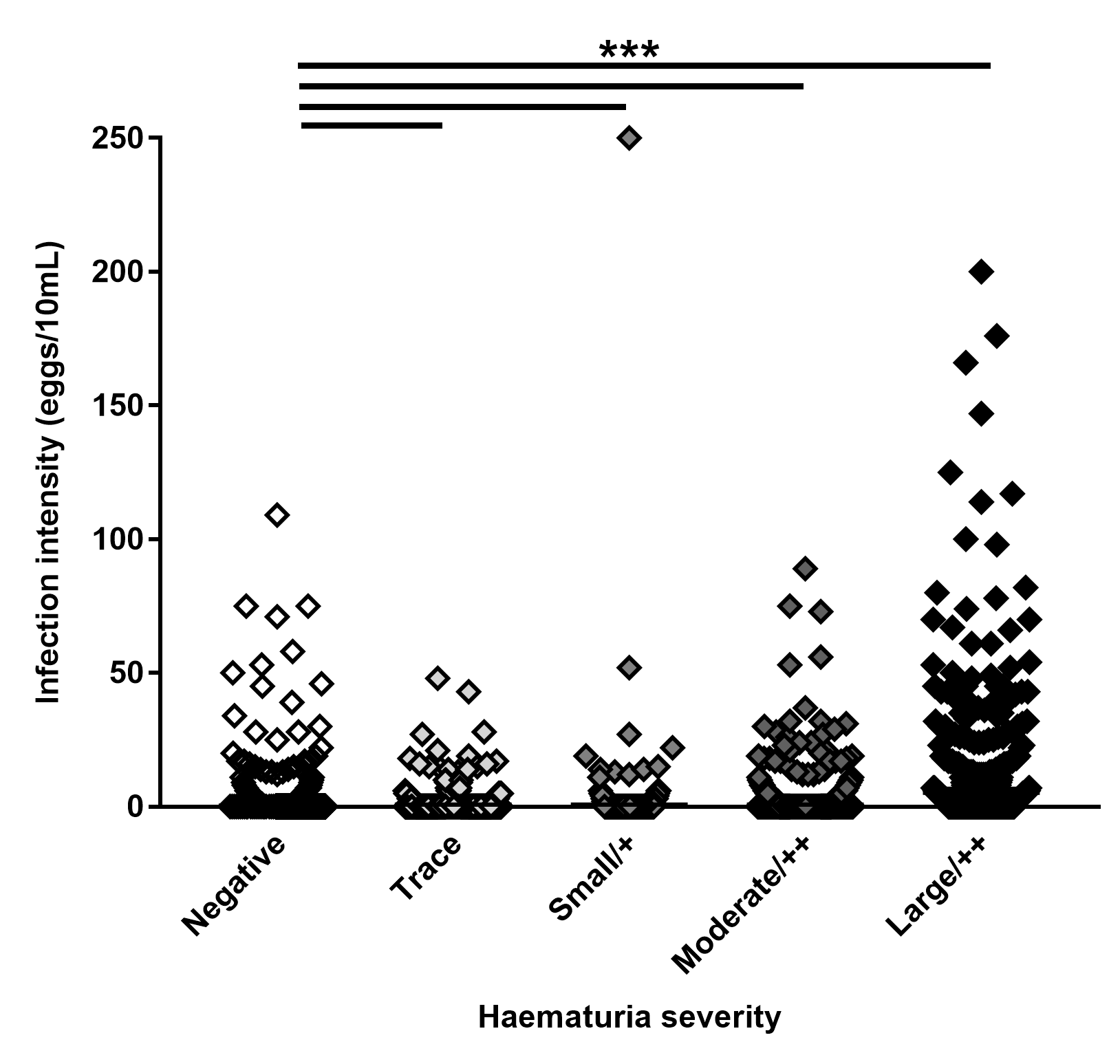


**Supplementary Table 1: Percentage of pregnant women with urogenital symptoms, anaemia and/or HIV**

| **Symptom** | **Egg+** | | | **Hematuria+** | | | **Egg+ *and/or*** **Hematuria+** | | |
| --- | --- | --- | --- | --- | --- | --- | --- | --- | --- |
|  | **Yes** | **No** | **P** | **Yes** | **No** | **P** | **Yes** | **No** | **P** |
|  | *n=467* | *n=3932* |  | *n= 1048* | *n= 3249* |  | *n=1151* | *n=3152* |  |
| **Vaginal discharge^a^** | 5.2% | 5.4% | 0.898 | 5.4% | 5.2% | 0.735 | 5.5% | 5.1% | 0.663 |
| **Genital warts^a^** | 3.9% | 2.4% | 0.063 | 3.4% | 2.3% | 0.053 | 3.2% | 2.4% | 0.128 |
| **STI^a^** | 2.1% | 2.0% | 0.818 | 1.7% | 2.1% | 0.417 | 1.7% | 2.1% | 0.423 |
| **Vaginal itch^a^** | 9.4% | 8.8% | 0.641 | 10.0% | 8.4% | 0.115 | 9.9% | 8.4% | 0.125 |
| **Vaginal bleeding^a^** | 3.4% | 2.3% | 0.130 | 2.6% | 2.4% | 0.705 | 2.6% | 2.4% | 0.625 |
| **Lower abdominal pain^a^** | 14.1% | 11.9% | 0.158 | 13.7% | 11.6% | 0.065 | 14.0% | 11.5% | 0.026 |
| **Mean Hb^b^,** *Mean (SD)* | 11.9 (1.64) | 12.0  (1.45) | 0.091 | 11.9  (1.59) | 12.0  (1.43) | 0.019 | 11.9  (1.59) | 12.0  (1.42) | 0.024 |
| **HIV status^a^,** *% positive* | 15.5% | 16.6% | 0.639 | 17.5% | 16.1% | 0.074 | 17.1% | 16.2% | 0.163 |

STI: sexually-transmitted infection; Hb: haemoglobin (g/L); SD: standard deviation

^a^Cluster-adjusted χ^2^ tests

^b^Two-sample t-test with equal variances

**Supplementary Table 2: Univariable general estimating equation logistic regression analysis of risk factors for being *S. haematobium* egg-positive or hematuria-positive among pregnant women**

|  | **GEE logistic regression model** | | | |
| --- | --- | --- | --- | --- |
| **Factor** | ***S. haematobium* egg+**  *n=4437* | | **Hematuria+**  *n=4298* | |
|  | **Unadj OR**^a,b^ **(95% CI)** | **P** | **Unadj OR**^a,c^ **(95% CI)** | **P** |
| **Maternal factors** |  |  |  |  |
| Parity | 0.82 (0.74, 0.91) | <0.001 | 0.92 (0.86, 1.00) | 0.045 |
| Education | 0.94 (0.90, 0.99) | 0.015 | 0.93 (0.89, 0.97) | <0.001 |
| Age of mother | 0.94 (0.92, 0.96) | <0.001 | 0.97 (0.96, 0.98) | <0.001 |
| Employed (No) | 1.13 (0.78, 1.63) | 0.520 | 1.06 (0.82, 1.37) | 0.644 |
| Married (No) | 1.35 (0.90, 2.06) | 0.160 | 1.62 (1.17, 2.22) | 0.003 |
| HIV status: |  |  |  |  |
| Positive | 1.00 |  | 1.00 |  |
| Negative | 1.10 (0.85, 1.42) | 0.463 | 0,92 (0.77, 1.09) | 0.337 |
| Unknown | 0.50 (0.05, 4.58) | 0.540 | 2.07 (0.77, 5.53) | 0.148 |
| Religion: |  |  |  |  |
| Apostolic | 1.00 |  | 1.00 |  |
| Other Christians^d^ | 0.79 (0.62, 0.99) | 0.045 | 0.84 (0.72, 0.98) | 0.030 |
| Other religions | 1.18 (0.82, 1.69) | 0.384 | 1.00 (0.75, 1.33) | 1.000 |
| **Household factors** |  |  |  |  |
| Household wealth: |  |  |  |  |
| Lowest quintile | 1.00 |  | 1.00 |  |
| Second | 0.92 (0.68, 1.25) | 0.592 | 0.92 (0.73, 1.16) | 0.484 |
| Middle | 0.75 (0.55,1.02) | 0.070 | 0.82 (0.66, 1.06) | 0.141 |
| Fourth | 0.72 (0.53, 0.98) | 0.036 | 0.77 (0.60, 0.98) | 0.037 |
| Highest quintile | 0.71 (0.52, 0.97) | 0.031 | 0.68 (0.53, 0.87) | 0.002 |
| Household size | 1.00 (0.95, 1.05) | 0.903 | 1.00 (0.97, 1.03) | 0.961 |
| **WASH factors** |  |  |  |  |
| Improved drinking water: |  |  |  |  |
| Yes | 1.00 |  | 1.00 |  |
| No | 1.23 (1.01, 1.49) | 0.044 | 1.25 (1.07, 1.46) | 0.006 |
| Any latrine: |  |  |  |  |
| Yes | 1.00 |  | 1.00 |  |
| No | 1.14 (0.93, 1.40) | 0.211 | 1.34 (1.15, 1.60) | <0.001 |
| Improved latrine: |  |  |  |  |
| Yes | 1.00 |  | 1.00 |  |
| No | 1.13 (0.91, 1.40) | 0.279 | 1.34 (1.14 , 1.58) | <0.001 |
| Handwashing station: |  |  |  |  |
| Yes | 1.00 |  | 1.00 |  |
| No | 1.12 (0.80, 1.57) | 0.495 | 1.04 (0.82, 1.31) | 0.743 |
| Handwashing station with water: |  |  |  |  |
| Yes | 1.00 |  | 1.00 |  |
| No | 0.94 (0.53, 1.67) | 0.835 | 0. 85 (0.55, 1.33) | 0.485 |
| Time to water | 1.00 (0.99, 1.00) | 0.579 | 1.00 (1.00, 1.00) | 0.683 |
| **Environmental factors** |  |  |  |  |
| Minimum temperature | 0.83 (0.38, 1.82) | 0.637 | 0.54 (0.30, 0.99) | 0.046 |
| Maximum temperature | 1.32 (0.77, 2.25) | 0.307 | 1.03 (0.68 , 1.57) | 0.881 |
| Mean rainfall | 1.00 (0.99, 1.00) | 0.554 | 1.00 (0.99, 1.01) | 0.892 |
| Season: |  |  |  |  |
| Rainy | 1.00 |  | 1.00 |  |
| Cold | 1.05 (0.81, 1.35) | 0.723 | 1.18 (0.99, 1.40) | 0.062 |
| Hot & dry | 1.02 (0.82, 1.27) | 0.878 | 1.32 (1.10, 1.58) | 0.002 |
| Field Office/Hub: |  |  |  |  |
| Chirumanzu district |  |  |  |  |
| Mvuma | 1.00 |  | 1.00 |  |
| St Theresa | 1.00 (0.70, 1.43) | 0.982 | 0.73 (0.57, 0.94) | 0.014 |
| Shurugwi district |  |  |  |  |
| Shurugwi | 1.62 (1.20, 2.19) | 0.002 | 1.99 (1.55, 2.56) | <0.001 |
| Tongogara | 1.03 (0.74,1.44) | 0.858 | 1.11 (0.87, 1.42) | 0.412 |

OR: Odds Ratio; IRR: Incidence Risk Ratio; CI: Confidence Interval

^a^Unadjusted

^b^Odds of being positive for ≥1 *S. haematobium* egg per 10mL urine

^c^Odds of being positive for hematuria

^d^Other Christians include: Protestants, Pentecostals, Catholics and other Christian groups

**Supplementary Table 3: Univariable zero-inflated beta regression analysis of risk factors for high *S. haematobium* infection intensity among pregnant women**

| **Factor** | **Zero-inflated negative binomial model** | | | |
| --- | --- | --- | --- | --- |
|  | **Odds of egg-negative**  *n=4437* | | **Infection intensity**  *n=4430^a^* | |
|  | **Unadj OR**^b,c^ **(95% CI)** | **P** | **IRR (95% CI)** | **P** |
| Parity |  |  |  |  |
| Education | 1.22 (1.10, 1.36) | <0.001 | 0.90 (0.77, 1.04) | 0.159 |
| Age of mother | 1.06 (1.01, 1.12) | 0.019 | 0.96 (0.89, 1.04) | 0.358 |
| Employed (No) | 1.07 (1.05, 1.09) | <0.001 | 0.99 (0.96, 1.02) | 0.451 |
| Married (No) | 0.92 (0.63, 1.33) | 0.655 | 1.05 (0.63, 1.74) | 0.857 |
| HIV status: | 0.76 (0.49, 1.18) | 0.220 | 1.34 (0.84, 2.13) | 0.221 |
| Positive | 1.00 |  | 1.00 |  |
| Negative | 0.91 (0.71, 1.17) | 0.448 | 0.89 (0.64, 1.26) | 0.524 |
| Unknown | 1.99 (0.24, 16.12) | 0.521 | 2.17 (1.56, 3.02) | <0.001 |
| Religion: |  |  |  |  |
| Apostolic | 1.00 |  | 1.00 |  |
| Other Christians^d^ | 1.30 (1.02, 1.64) | 0.033 | 1.24 (0.90, 1.72) | 0.188 |
| Other religions | 0.77 (0.54, 1.11) | 0.165 | 0.76 (0.50, 1.14) | 0.189 |
| **Household factors** |  |  |  |  |
| Household wealth: |  |  |  |  |
| Lowest quintile | 1.00 |  | 1.00 |  |
| Second | 1.12 (0.82, 1.52) | 0.488 | 1.20 (0.81, 1.78) | 0.360 |
| Middle | 1.37 (1.00, 1.88) | 0.047 | 0.99 (0.70, 1.39) | 0.947 |
| Fourth | 1.43 (1.05, 1.95) | 0.025 | 1.08 (0.71, 1.63) | 0.720 |
| Highest quintile | 1.48 (1.09, 2.00) | 0.012 | 1.10 (0.75, 1.62) | 0.627 |
| Household size | 1.00 (0.95, 1.06) | 0.866 | 1.03 (0.96, 1.11) | 0.364 |
| **WASH factors** |  |  |  |  |
| Improved drinking water: |  |  |  |  |
| Yes | 1.00 |  | 1.00 |  |
| No | 0.80 (0.65, 0.97) | 0.026 | 1.04 (0.76, 1.44) | 0.795 |
| Any latrine: |  |  |  |  |
| Yes | 1.00 |  | 1.00 |  |
| No | 0.87 (0.71, 1.08) | 0.214 | 1.06 (0.77 ,1.48) | 0.708 |
| Improved latrine: |  |  |  |  |
| Yes | 1.00 |  | 1.00 |  |
| No | 0.87 (0.70, 1.09) | 0.220 | 1.01 (0.72, 1.41) | 0.954 |
| Handwashing station: |  |  |  |  |
| Yes | 1.00 |  | 1.00 |  |
| No | 0.98 (0.72, 1.34) | 0.910 | 1.04 (0.66, 1.65) | 0.860 |
| Handwashing station with water: |  |  |  |  |
| Yes | 1.00 |  | 1.00 |  |
| No | 1.17 (0.68, 2.01) | 0.570 | 0.87 (0.44, 1.73) | 0.701 |
| Time to water | 1.00 (1.00, 1.01) | 0.679 | 1.00 (0.99, 1.01) | 0.969 |
| **Environmental factors** |  |  |  |  |
| Minimum temperature | 1.65 (0.66, 4.11) | 0.283 | 1.75 (0.68, 4.51) | 0.244 |
| Maximum temperature | 0.89 (0.50, 1.60) | 0.703 | 0.86 (0.40, 1.83) | 0.691 |
| Mean rainfall | 1.00 (0.99, 1.01) | 0.758 | 1.01 (1.00, 1.01) | 0.209 |
| Season: |  |  |  |  |
| Rainy | 1.00 |  | 1.00 |  |
| Cold | 0.93 (0.72, 1.21) | 0.601 | 1.05 (0.73, 1.51) | 0.780 |
| Hot & dry | 0.98 (0.78, 1.24) | 0.888 | 0.97 (0.68, 1.37) | 0.864 |
| Field Office/Hub: |  |  |  |  |
| Chirumanzu district |  |  |  |  |
| Mvuma | 1.00 |  | 1.00 |  |
| St Theresa | 1.00 (0.69, 1.46) | 0.979 | 0.91 (0.59, 1.39), | 0.656 |
| Shurugwi district |  |  |  |  |
| Shurugwi | 0.60 (0.43, 0.82) | 0.002 | 0.62 (0.43, 0.91) | 0.014 |
| Tongogara | 1.01 (0.72, 1.43) | 0.933 | 1.34 (0.86, 2.10) | 0.197 |

OR: Odds Ratio; IRR: Incidence Risk Ratio; CI: Confidence Interval

^a^7 participants had known schistosomiasis status but unknown infection intensity

^b^Unadjusted

^c^Odds of being negative for *S. haematobium* eggs in urine

^d^Other Christians include: Protestants, Pentecostals, Catholics and other Christian groups

**Supplementary Table 4: Prevalence of urogenital schistosomiasis in the case-control study of adverse birth outcomes among pregnant women**

| **Adverse Outcome** | **Cases** | | | | **Controls** | | | | |
| --- | --- | --- | --- | --- | --- | --- | --- | --- | --- |
|  | ***N*** | ***Egg+*** | ***Hematuria+*** | ***Egg &/or***  ***Hematuria+*** |  | ***n*** | ***Egg+*** | ***Hematuria+*** | ***Egg &/or***  ***Hematuria+*** |
| **Miscarriage**^a^ | 132 | 9.8% | 23.8% | 26.9% |  | 264 | 13.6% | 31.0% | 33.7% |
| **Stillbirth**^a,b^ | 79 | 5.1% | 23.4% | 26.0% |  | 158 | 7.0% | 22.4% | 24.4% |
| **SGA/Term**^a,b^ | 478 | 12.3% | 27.9% | 29.1% |  | 956 | 9.8% | 24.2% | 26.5% |
| **SGA/Preterm**^a,b^ | 30 | 20.0% | 30.0% | 36.7% |  | 60 | 5.0% | 21.7% | 21.7% |
| **AGA/Preterm**^a,b^ | 495 | 12.3% | 21.9% | 25.7% |  | 990 | 13.1% | 24.4% | 28.7% |
| **Neonatal death**^a,b^ | 62 | 11.3% | 16.7% | 21.7% |  | 124 | 14.3% | 23.2% | 26.4% |

SGA – small-for-gestational age; AGA – appropriate-for-gestational age

^a^Cases and controls were matched on maternal HIV status, gestational age at the baseline visit (±2 weeks) and SHINE study arm

^b^Cases and controls were also matched on infant sex

**Supplementary Tables 5: Unadjusted association between maternal *S. haematobium* infection intensity during pregnancy and adverse birth outcomes**

| **Infection intensity** | **Miscarriage** | | | **Stillbirth** | | | **SGA Term** | | |
| --- | --- | --- | --- | --- | --- | --- | --- | --- | --- |
|  | **n**  **Cases, Controls** | **OR^a^**  **(95% CI)** | **P** | **n**  **Cases, Controls** | **OR^a^**  **(95% CI)** | **P** | **n Cases, Controls** | **OR^a^**  **(95% CI)** | **P** |
| ***Negative***  *<1egg/10mL* | 119, 230 | 1.00 |  | 75, 147 | 1.00 |  | 419, 862 | 1.00 |  |
| ***Low***  *1-50eggs/10mL* | 13, 28 | 0.91  (0.46, 1.78) | 0.778 | 4, 10 | 0.79  (0.24, 2.61) | 0.696 | 54, 87 | 1.28  (0.89, 1.84) | 0.176 |
| ***High***  *>50eggs/10mL* | 0, 3 | ^b^ | ^b^ | 0, 0 | ^b^ | ^b^ | 5, 7 | 1.49  (0.88, 0.50) | 0.495 |

| **Infection intensity** | **SGA Preterm** | | | **AGA Preterm** | | | **Neonatal death** | | |
| --- | --- | --- | --- | --- | --- | --- | --- | --- | --- |
|  | **n**  **Cases, Controls** | **OR^a^**  **(95% CI)** | **P** | **n**  **Cases, Controls** | **OR^a^**  **(95% CI)** | **P** | **n**  **Cases, Controls** | **OR^a^**  **(95% CI)** | **P** |
| ***Negative***  *<1egg/10mL* | 24, 57 | 1.00 |  | 434, 860 | 1.00 |  | 55, 108 | 1.00 |  |
| ***Low***  *1-50eggs/10mL* | 5, 3 | 4.33  (0.82, 22.82) | 0.084 | 57, 115 | 0.96  (0.68, 1.36) | 0.831 | 6, 16 | 0.75  (0.27, 2.10) | 0.585 |
| ***High***  *>50eggs/10mL* | 1, 0 | ^b^ | ^b^ | 3, 12 | 0.50  (0.14, 1.76) | 0.278 | 1, 2 | 1.23  (0.07, 22.30) | 0.891 |

OR: Odds Ratio; CI: Confidence Interval

^a^Unadjusted conditional logistic regression models

^b^Too few women with high intensity infections within the case control cohort to assess associations with the adverse birth outcome

**Supplementary Tables 6: Unadjusted association between the severity of maternal hematuria during pregnancy and adverse birth outcomes**

| **Hematuria severity** | **Miscarriage** | | | **Stillbirth** | | | **SGA Term** | | |
| --- | --- | --- | --- | --- | --- | --- | --- | --- | --- |
|  | **n Cases, Controls** | **OR^a^**  **(95% CI)** | **P** | **n Cases, Controls** | **OR^a^**  **(95% CI)** | **P** | **n Cases, Controls** | **OR^a^**  **(95% CI)** | **P** |
| ***Negative*** | 99, 176 | 1.00 |  | 59, 121 | 1.00 |  | 339, 710 | 1.00 |  |
| ***Trace*** | 11, 26 | 0.76  (0.35, 1.64) | 0.486 | 5, 15 | 0.68  (0.24, 1.94) | 0.470 | 40, 92 | 0.90  (0.61, 1.33) | 0.605 |
| ***Small***  ***(+)*** | 1, 14 | 0.16  (0.02, 1.22) | 0.077 | 3, 5 | 1.20  (0.28, 5.14) | 0.804 | 15, 19 | 1.73  (0.86, 3.49) | 0.125 |
| ***Moderate (++)*** | 11, 19 | 1.10  (0.51, 2.39) | 0.801 | 8, 6 | 2.67  (0.85, 8.37) | 0.093 | 39, 60 | 1.37  (0.89, 2.11) | 0.155 |
| ***Large (+++)*** | 8, 20 | 0.67  (0.28, 1.62) | 0.376 | 2, 9 | 0.54  (0.11, 2.59) | 0.445 | 37, 56 | 1.36  (0.88, 2.10) | 0.172 |

| **Hematuria severity** | **SGA Preterm** | | | **AGA Preterm** | | | **Neonatal death** | | |
| --- | --- | --- | --- | --- | --- | --- | --- | --- | --- |
|  | **n Cases, Controls** | **OR^a^**  **(95% CI)** | **P** | **n Cases, Controls** | **OR^a^**  **(95% CI)** | **P** | **n Cases, Controls** | **OR^a^**  **(95% CI)** | **P** |
| ***Negative*** | 21, 47 | 1.00 |  | 370, 739 | 1.00 |  | 50, 96 | 1.00 |  |
| ***Trace*** | 3, 4 | 1.34  (0.26, 6.98) | 0.730 | 44, 94 | 0.93  (0.63, 1.36) | 0.695 | 2, 12 | 0.36  (0.08, 1.72) | 0.200 |
| ***Small***  ***(+)*** | 0, 3 | - | - | 16, 16 | 1.77  (0.87, 3.57) | 0.113 | 2, 4 | 1.00  (0.16, 6.42) | 1.000 |
| ***Moderate (++)*** | 1, 6 | 0.36  (0.04, 3.11) | 0.350 | 15, 81 | 0.35  (0.20, 0.62) | <0.001 | 1, 6 | 0.42  (0.05, 3.63) | 0.430 |
| ***Large (+++)*** | 5, 0 | ^b^ | ^b^ | 29, 48 | 1.23  (0.76, 2.00) | 0.390 | 5, 7 | 1.32  (0.40, 4.41) | 0.651 |

OR: Odds Ratio; CI: Confidence Interval

^a^Unadjusted conditional logistic regression models

^b^Too few women with Large/+++ hematuria within the case control cohort to assess associations with the adverse birth outcome

| **Infection Intensity** | **Birthweight (g)** *n=3667* | | **1 month LAZ** *n=2275* | | | **18 month LAZ**  *n=3752* | |
| --- | --- | --- | --- | --- | --- | --- | --- |
|  | **Coeff**^a^ **(95% CI)** | **P** | **Coeff**^a^  **(95% CI)** | **P** | | **Coeff**  **(95% CI)** | **P** |
| ***Negative***  *<1egg/10mL* | 1.00 |  | 1.00 | |  | 1.00 |  |
| ***Low***  *1-50eggs/10mL* | -0.05  (-0.10, 0.01) | 0.081 | 0.10  (-0.11, 0.31) | | 0.339 | -0.07  (-0.19, 0.05) | 0.257 |
| ***High***  *>50eggs/10mL* | -0.05  (-0.25, 0.14) | 0.582 | 0.22  (-0.42, 0.85) | | 0.502 | -0.11  (-0.39, 0.17) | 0.439 |

**Supplementary Tables 7: Unadjusted association between maternal *S. haematobium* infection intensity during pregnancy and child growth outcomes**

| **Infection Intensity** | **Low birthweight**  *n=3667* | | **1 month stunted**  *n=2275* | | **18 month stunted**  *n=3757* | | | | |
| --- | --- | --- | --- | --- | --- | --- | --- | --- | --- |
|  | **OR**^a^  **(95% CI)** | **P** | **OR**^a^  **(95% CI)** | **P** | | **OR**^a^ **(95% CI)** |  | **P** |  |
| ***Negative***  *<1egg/10mL* | 1.00 |  | 1.00 |  | 1.00 | |  | | |
| ***Low***  *1-50eggs/10mL* | 1.36  (0.92, 2.01) | 0.118 | 0.72  (0.46, 1.15) | 0.172 | 1.04  (0.81, 1.32) | | 0.778 | | |
| ***High***  *>50eggs/10mL* | 0.09  (0.08, 0.10) | 0.776 | 0.77  (0.17, 3.43) | 0.727 | 1.18  (0.54, 2.60) | | 0.674 | | |

Coeff: Coefficient; CI: Confidence Interval; OR: Odds Ratio

^a^Unadjusted GEE population-averaged model

**Supplementary Tables 8: Unadjusted association between the severity of maternal hematuria during pregnancy and child growth outcomes**

| **Hematuria severity** | **Birthweight (g)**  *n=3563* | | **1 month LAZ**  *n=2217* | | **18 month LAZ** *n=3648* | |
| --- | --- | --- | --- | --- | --- | --- |
|  | **Coeff**^a^  **(95% CI)** | **P** | **Coeff**^a^  **(95% CI)** | **P** | **Coeff**^a^  **(95% CI)** | **P** |
| ***Negative*** | 1.00 |  | 1.00 |  | 1.00 |  |
| ***Trace*** | 0.02  (-0.04, 0.07) | 0.554 | -0.12  (-0.32, 0.08) | 0.248 | 0.03  (-0.10, 0.15) | 0.682 |
| ***Small***  ***(+)*** | -0.06  (-0.17, 0.04) | 0.226 | -0.29  (-0.67, 0.08) | 0.127 | -0.13  (-0.38, 0.11) | 0.279 |
| ***Moderate (++)*** | 0.00  (-0.06, 0.07) | 0.976 | 0.04  (-0.20, 0.27) | 0.765 | -0.06  (-0.21, 0.09) | 0.441 |
| ***Large (+++)*** | -0.06  (-0.12, 0.01) | 0.087 | 0.01  (-0.24, 0.26) | 0.950 | -0.12  (-0.27, 0.03) | 0.108 |

| **Hematuria severity** | **Low birthweight**  *n=3563* | | **1 month stunted**  *n=2217* | | **18 month stunted**  *n=3653* | |
| --- | --- | --- | --- | --- | --- | --- |
|  | **OR**^a^  **(95% CI)** | **P** | **OR**^a^  **(95% CI)** | **P** | **OR**^a^ **(95% CI)** | **P** |
| ***Negative*** | 1.00 |  | 1.00 |  | 1.00 |  |
| ***Trace*** | 0.96  (0.60, 1.54) | 0.875 | 0.86  (0.55, 1.32) | 0.480 | 1.10 (0.86, 1.42) | 0.440 |
| ***Small***  ***(+)*** | 1.74  (0.86, 3.50) | 0.123 | 1.76  (0.96, 3.21) | 0.067 | 1.13 (0.74, 1.72) | 0.572 |
| ***Moderate (++)*** | 1.01  (0.60, 1.72) | 0.956 | 1.18  (0.72, 1.92) | 0.517 | 1.03 (0.76, 1.39) | 0.869 |
| ***Large (+++)*** | 1.10  (0.66, 1.84) | 0.716 | 0.74  (0.42, 1.30) | 0.295 | 1.07 (0.81, 1.41) | 0.651 |

Coeff: Coefficient; CI: Confidence Interval; OR: Odds Ratio

^a^Unadjusted GEE population-averaged model
