# Supplementary figures and images for: Determinants of Urogenital Schistosomiasis Among Pregnant Women and its Association With Pregnancy Outcomes, Neonatal Deaths, and Child Growth
Source: J Infect Dis. 2019 Dec 13;223(8):1433–44. doi: 10.1093/infdis/jiz664 (PMC8064048; doi:10.1093/infdis/jiz664)

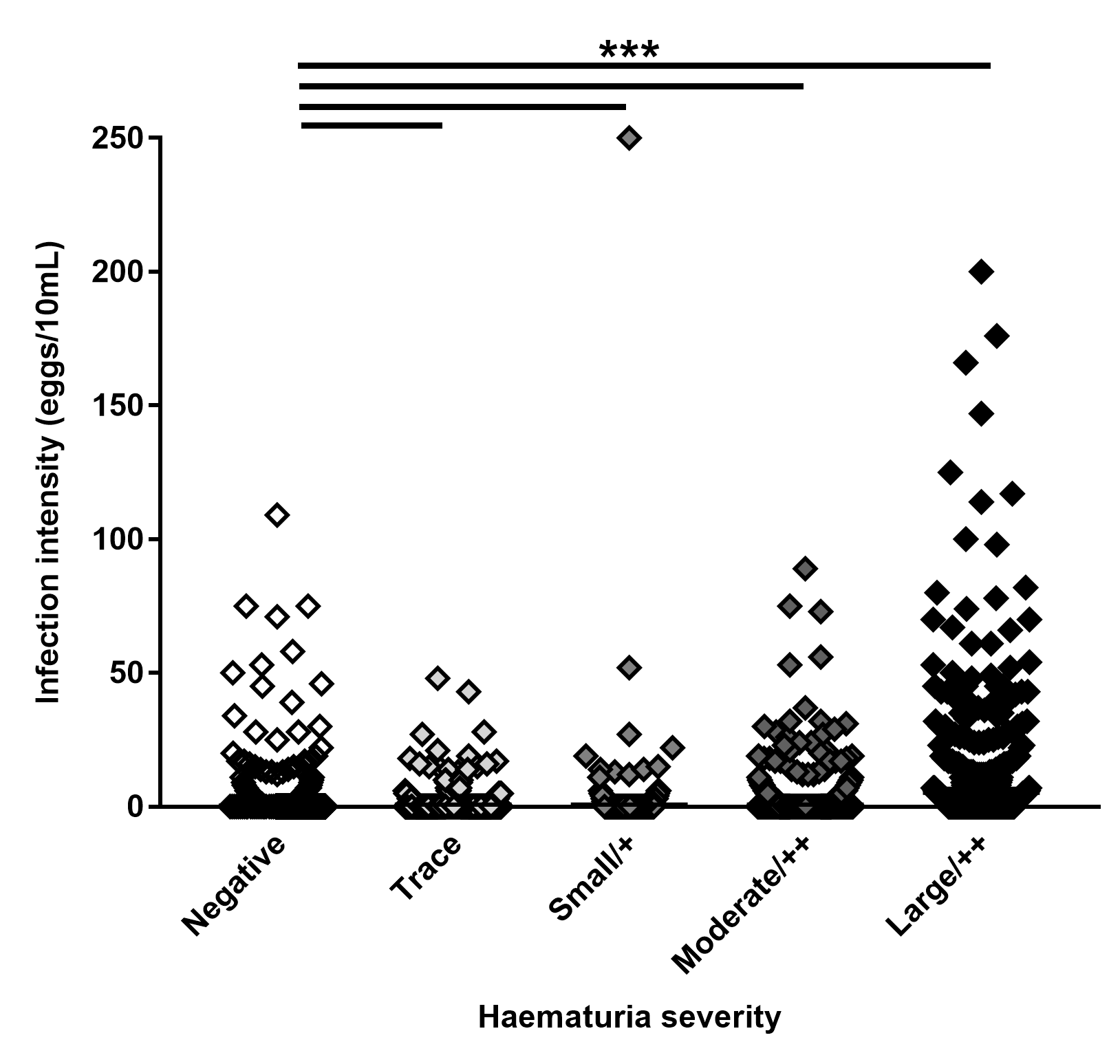

Supplement: jiz664_suppl_Supplementary_Figure_S1 [file jiz664_suppl_supplementary_figure_s1.png]
